# Antibiotic Resistance of Bacteria Isolated from the Internal Organs of Edible Snow Crabs

**DOI:** 10.1371/journal.pone.0070887

**Published:** 2013-08-21

**Authors:** Misoon Kim, Tae-Hyung Kwon, Su-Mi Jung, Seung-Hak Cho, Seon Yeong Jin, Nyun-Ho Park, Choong-Gon Kim, Jong-Shik Kim

**Affiliations:** 1 Gyeongbuk Institute for Marine Bioindustry, Jukbyeon-Meon, Uljin-Gun, Gyeongbuk, Republic of Korea; 2 Division of Enteric Bacterial Infections, Center for Infectious Diseases, Korea National Institute of Health, Cheongwon-Gun, Chungcheongbuk, Republic of Korea; Beijing Institute of Microbiology and Epidemiology, China

## Abstract

Antibiotic resistance and microbiota within edible snow crabs are important for the *Chionoecetes* (snow crab) fishing industry. We investigated these parameters using culture methods and antibiotic susceptibility tests with six internal organs from three species of *Chionoecetes*. Each sample revealed many unexpected microbial species within *Chionoecetes* internal organs. On the basis of 16S rRNA sequence analysis of 381 isolates, the most abundant genera identified in *Chionoecetes opilio* were *Acinetobacter* spp. (24%), *Bacillus* spp. (4%), *Pseudomonas* spp. (34%), *Stenotrophomonas* spp. (28%), and *Agreia* spp. (11%). In *Chionoecetes* sp. crabs, *Acinetobacter* spp. (23%), *Bacillus* spp. (12%), and *Psychrobacter* spp. (20%) were most prevalent, while *Agreia* spp. (11%), *Bacillus* spp. (31%), *Microbacterium* spp. (10%), *Rhodococcus* spp. (12%), and *Agrococcus* spp. (6%) were most abundant in *C. japonicus*. Our antibiotic resistance test found resistance to all nine antibiotics tested in 19, 14, and two of the isolates from *C. opilio*, *Chionoecetes* sp., and, *C. japonicus* respectively. Our results are the first to show that microbes with antibiotic resistance are widely distributed throughout the internal organs of natural snow crabs.

## Introduction

Snow crabs belong to the subphylum Crustacea, Order Decapoda, Family Majidae, and Genus *Chionoecetes*. These *Chionoecetes* are found in colder water at depths less than 2000 m where it is muddy or sandy [1]. Three kinds of *Chionoecetes* are prominent on the east coast of Korea, including snow crab (*Chionoecetes opilio*), red-tanner crab (*Chionoecetes japonicus*), and the hybrid Neodo-Daege (*Chionoecetes* sp.) [2]. *Chionoecetes* fishing is a major industry and source of income in the area [3]–[6]. However, contamination of the East Sea of Korea from human activities has raised serious sanitation concerns that could potentially threaten this industry [7]. Research on antibiotic resistance has primarily focused on human disease; there is limited understanding of antibiotic resistance genes in natural environments. The relationship between environmental microorganisms and human pathogens is not clear; a recent report showed that soil bacteria and human pathogens shared an antibiotic resistome [8]. In this study, we analyzed the microbiota within parts of *Chionoecetes* using culturing methods, and the culturable microbial isolates were tested for antibiotic resistance. Here, we report the microbial populations and antibiotic resistance of isolates from the internal organs of *Chionoecetes*. These results may be used to monitor snow crab populations and to identify potentially dangerous changes in microbiota that could threaten the snow crab industry.

## Materials and Methods

### Collection of snow crabs

No specific permissions were required for these locations or activities because many snow crabs were sold daily at the market near the Harbor. Wild-caught, uncooked snow crabs (*Chionoecetes* spp.) were collected from a retail seafood shop at Jukbyeon Harbor and were placed into clean, resealable plastic bags. Samples were stored in a cooler during transfer from Jukbyeon Harbor to the laboratory where they were then stored at 4°C until processed. The morphological characteristics of snow crabs can be distinguished by their carapace color, the arrangement of granules on the lateral carapace, and the presence or absence of spines on the lateral carapace [4]. The crabs were originally caught in the East Sea of Korea [5], [6]. There are no-take periods from June through November for snow crab (*Chionoecetes opilio*) and July through August for red-tanner crab (*Chionoecetes japonicus*), and no prohibition for Neodo-Daege (*Chionoecetes* sp.) for management of the snow crab industry.

### Sample preparation

Samples were divided into the following six parts: guts (D), gills (G), heart (H), leg meat (LS), carapace meat (S), and carapace juices (J). Each sample was homogenized with 10 mL of 10 mM potassium phosphate buffer, and 100 µL of each sample was spread onto agar plates.

### Enumeration of microbial populations

The serial dilutions were spread plated on various media to determine microbial counts of the *Chionoecetes* spp. in sterile water, and the dilutions were dispensed onto agar plates. For all experiments reported herein, we cultured aerobic microbes on the following media: YPD (yeast extract, peptone, dextrose, BD Bioscience, USA) with chloramphenicol (100 mg/L) and streptomycin (100 mg/L) for yeast; PDA (potato dextrose agar, BD Bioscience) with chloramphenicol (100 mg/L) and streptomycin (100 mg/L) for fungi; R2A (BD Bioscience); NA (nutrient agar, BD Bioscience); TSA (tryptic soy agar, BD Bioscience) for general bacteria; or MA (marine agar, BD Bioscience). The plates were incubated aerobically at 28°C for 1 wk, and average CFU (colony-forming units) values were obtained from triplicate plate counts.

### Isolation of bacteria

All colonies from individual plates of one plate or two plates from the plates of colony counts were picked up and cultured separately. In total, 381 individual isolates were transferred to fresh plates three times and then processed for sequencing of 16S rRNA and ITS genes.

### Sequencing

The primers used to amplify the 16S rRNA and ITS genes for bacteria and other microbes were 27F and 1492R [9] and ITS1 and ITS4 [10], respectively. The PCR reaction was performed with 20 ng of genomic DNA as the template in a 30-µL reaction mixture by using EF-*Taq* DNA polymerase (Solgent, Korea). The thermocycler conditions were 95°C for 5 min, followed by 35 cycles of 95°C for 2 min, 55°C for 60 s, and 72°C for 60 s, then a final extension step for 10 min at 72°C. Thereafter, the amplification products were purified using a multiscreen filter plate (Millipore Corp., Bedford, MA, USA). Sequencing reactions were performed using a PRISM BigDye Terminator v3.1 Cycle Sequencing Kit (Applied Bio- systems, Foster City, Calif., USA). The DNA samples containing the extension products were added to Hi-Di formamide (Applied Biosystems, Foster City, CA). The mixture was incubated at 95°C for 5 min followed by 5 min on ice, and then analyzed using an ABI Prism 3730XL DNA analyzer (Applied Biosystems, Foster City, CA). DNA sequencing of isolates was performed by Macrogen Inc. (Seoul, Korea).

### Phylogenetic trees

The 16S rRNA and ITS sequences were aligned using the Nearest Alignment Space Termination (NAST) aligner [11]. Aligned sequences were then compared to the Lane mask using the Greengenes website [12]. Sequence matching to the Ribosomal Database Project [13] was used to find GenBank sequences representing the most closely related type of strain for each isolate. These type strains were included as references in the phylogeny using the Greengenes Automatic Taxonomic Classification algorithm [12]. Phylogenetic trees were constructed using neighbor-joining [14] with MEGA5 for Windows [15]. Evolutionary distances were calculated with the Kimura 2-parameter method. Bootstrap analyses of the neighbor-joining data were conducted based on 1000 samples to assess the support for inferred phylogenetic relationships.

### Antibiotics

The antibiotics (content per disc) used in the study were ampicillin (10 µg), chloramphenicol (30 µg), erythromycin (15 µg), kanamycin (30 µg), penicillin (10 unit), rifampicin (5 µg), tetracyclin (30 µg), ticarcilin (75 µg), and vancomycin (30 µg). The antibiotic discs were purchased from BD Bioscience (San Jose, CA).

### Antibiotic susceptibility test

Bacteria were considered susceptible to a particular antibiotic if the bacteria formed a clear zone around a disc on the media (disc diffusion susceptibility testing). After autoclaving the nutrient agar and cooling the agar to 50–55°C, bacterial colonies were mixed into the autoclave medium flask and poured onto petri dishes. Each antibiotic susceptibility testing disc (BD Bioscience) was placed onto a plate and incubated at 28°C for 24 or 48 h. The results indicated whether the isolates were resistant or susceptible to each antibiotic.

### PCR assays for detection of resistance genes and sequencing of the PCR products

Bacteria were tested through PCR method with the primers of antibiotic resistance genes as shown in Table 1. For the PCR, the reaction mixtures contained 10 µl 2× DNA polymerase enzyme (PowerAmp™ 2× premix), 4 µl primer mixtures, 5 µl template DNA and sterile distilled water to bring the final volume to 20 µl. The PCR was performed with TaKaRa PCR Thermal Cycler Dice TP600 (TaKaRa, Japan). The reaction was started with a 15-min denaturation step at 95°C. In the PCRs, the temperature cycles consisted of 30 sec at 95°C, followed by 1 min at 58°C and 1 min at 72°C and each cycle was repeated 35 times. The final cycle was followed by incubation of the reaction mixture for 10 min at 72°C. Amplified PCR products were analyzed by gel electrophoresis in 2% agarose gels stained with ethidium bromide, visualized with ultraviolet illumination, and imaged with the Gel Doc 2000 documentation system (Bio-Rad, Hercules, CA, USA). DNA sequencing of antibiotic resistance genes was performed by Macrogen Inc. (Seoul, Korea).

**Table 1:** PCR primers targeting antibiotic resistance genes.

Antibiotics	Target gene		Sequence 5′-3′	Amplicon size (bp)	References
ampicillin	*blaSHV*	FW*	TTA TCT CCC TGT TAG CCA CC	796	[19]
		RV	GAT TTG CTG ATT TCG CTC GG		
	*blaOXA*	FW	ACC AGA TTC AAC TTT CAA	589	
		RV	TCT TGG CTT TTA TGC TTG		
	*blaTEM*	FW	ATA AAA TTC TTG AAG AC	1,073	
		RV	TTA CCA ATG CTT AAT CA		
chloramphenicol	*catA1*	FW	CGC CTG ATG AAT GCT CAT CCG	456	[17]
		RV	CCT GCC ACT CAT CGC AGT AC		
	*catA2*	FW	ATG AAT TTT ACC AGA ATT GAT CTG AA	639	
		RV	ATT TCA GTA TGT TAT CAC ACA TCA TCT		
	*catA3*	FW	AAA TTG GGT TCG CCG TGA	1,863	
		RV	ATT TAC TGT TAC ACA ACT CTT GTA GCC		
	*catB3*	FW	TCA AAG GCA AGC TGC TTT CTG AGC	566	
		RV	TAT TAG ACG AGC ACA GCA TGG GCA		
erythromycin	*ermA*	FW	TAT CTT ATC GTT GAG AAG GGA TT	138	[18]
		RV	CTA CAC TTG GCT TAG GAT GAA A		
	*ermB*	FW	CTA TCT GAT TGT TGA AGA AGG ATT	141	
		RV	GTT TAC TCT TGG TTT AGG ATG AAA		
	*mefA*	FW	AGT ATC ATT AAT CAC TAG TGC	348	[19]
		RV	TTC TTC TGG TAC TAA AAG TGG		
penicillin	*pbp2a*	FW	CCG CTG ATC TTG ATT GAA TAG	355	[20]
		RV	ATG CGT TTT CAT CCC CTC TG		
kanamycin	*aphA-3*	FW	GGGACCACCTATGATGTGGAACG	600	[21]
		RV	CAGGCTTGATCCCCAGTAAGTC		
tetracycline	*tetA*	FW	GTA ATT CTG AGC ACT GTC GC	956	[16]
		RV	CTG CCT GGA CAA CAT TGC TT		
	*tetB*	FW	ACG TTA CTC GAT GCC AT	1,169	
		RV	AGC ACT TGT CTC CTG TT		
	*tetC*	FW	AAC AAT GCG CTC ATC GT	1,138	
		RV	GGA GGC AGA CAA GGT AT		
	*tetG*	FW	CCG GTC TTA TGG GTG CTC TA	603	
		RV	CCA GAA GAA CGA AGC CAG TC		
vancomycin	*vanA*	FW	GCT ATT CAG CTG TAC TC	783	[22]
		RV	CAG CGG CCA TCA TAC GG		
	*vanB*	FW	CAT CGC CGT CCC CGA ATT TCA AA	297	
		RV	GAT GCG GAA GAT ACC GTG GCT		

* FW, forward; RV, reverse.

## Results

### Culturable microbiota

**Figure 1:**
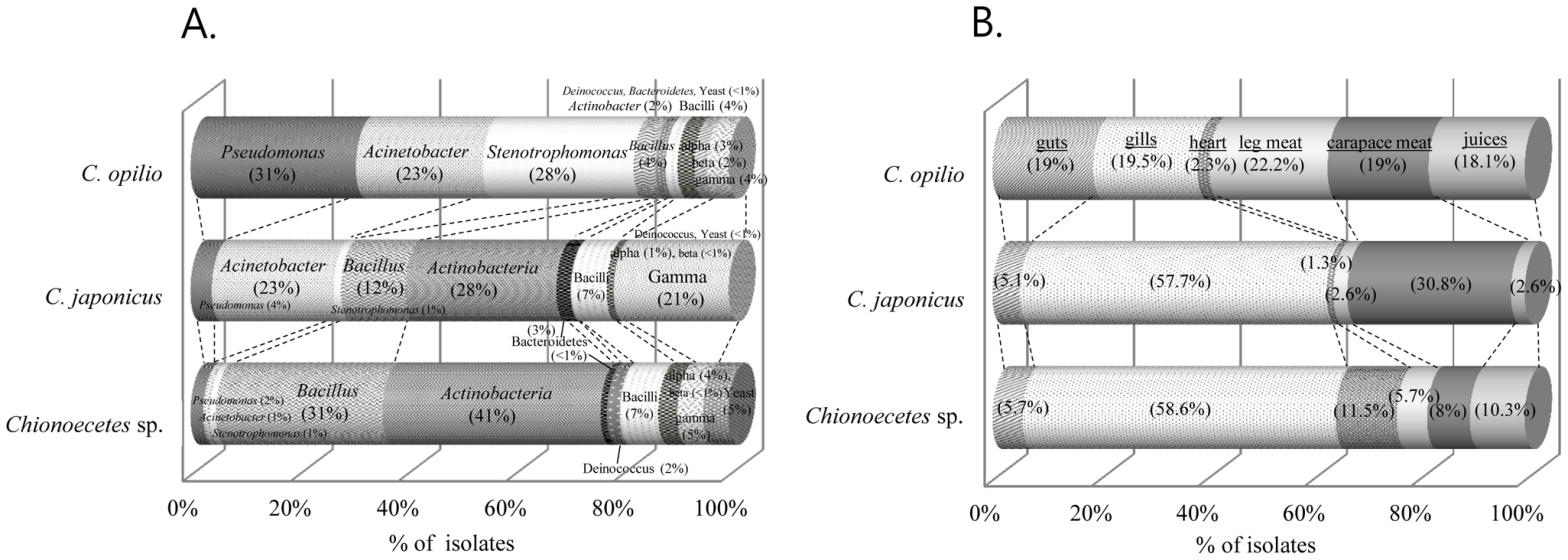
Stacked bar graphs of each phylum of three species of snow crabs (A), and isolates from each internal organ (B) in the three crab species.

A total of 381 isolates, including 221 from *C. opilio*, 76 from *C. japonicus*, and 84 from *Chionoecetes* sp. were isolated using six different media (Figure 1A). Figure 1 summarizes the phylogenetic distribution of the 16S rRNA gene sequences. The genera *Pseudomonas*, *Stenotrophomonas*, and *Acinetobacter* predominated the bacterial communities found in *Chionoecetes*, commonly representing more than 60% of the sequences isolated from the three crab species. The microbial isolates from *C. japonicus* and *C. opilio* included 19 and 20 genera, respectively, whereas the isolates of *Chionoecetes* sp. included 21 genera (Figure 1A). The greatest diversity of microbiota among the six different internal organs investigated was found in the gills; we identified 14, 15, and 17 genera in gills of *C. japonicus*, *C. opilio*, and *Chionoecetes* sp. respectively. Nine or fewer genera were identified in the other organs (Figures 1B and S1).

Isolates from snow crab were predominantly *Acinetobacter* spp. (Figure S1b), *Pseudomonas* spp. (Figure S1a), *Bacillus* spp. (Figure S1d), and *Stenotrophomonas* spp. (Figure S1e), which collectively accounted for approximately 84% of the isolates (n = 186). Forty-six isolates from *C. japonicus* included either *Acinetobacter* spp. (Figure S1b), *Agreia* spp. (Figure S1f), *Bacillus* spp. (Figure S1d), or *Psychrobacter* spp. (Figure S1f), whereas 60% of isolates (n = 57) from *Chionoecetes* sp. were affiliated with *Agreia* spp., *Agrococcus* spp. (Figure S1g), *Bacillus* spp., *Microbacterium* spp. (Figure S1g), and *Rhodococcus* spp. (Figure S1g). *Bacillus* spp. were common in all three species of snow crabs. *Pseudomonas* spp. (n = 68 isolates) were predominantly found in *C. opilio*, while only three and two bacterial species were isolated from the other two *Chionoecetes* spp. (Figures 1 and S1a). *Acinetobacter* spp. were predominant in *C. opilio* and *C. japonicus* (53 and 18 isolates, respectively), and only one bacterial isolate was found in *Chionoecetes* sp. (Figures 1 and S1b). *Stenotrophomonas* spp. dominated in *C. opilio* (62 isolates), while only one bacterial species from this genus was isolated from each of *C. japonicus* and *Chionoecetes* sp. (Figures 1 and S1c). Numbers of *Bacillus* spp. isolated included nine from *C. opilio*, nine from *C. japonicus*, and 26 from *Chionoecetes* sp. (Figures 1 and S1d).

In *C. opilio*, 67% (nine isolates) of *Bacillus* spp. were localized in the heart, while 33% (nine isolates) were found in the gill and carapace in *C. japonicus*. *Bacillus* spp. from *Chionoecetes* sp. were found in the carapace juices and heart (26% and 30% of 26 isolates respectively). Some bacterial isolates were confined to particular parts of *Chionoecetes*. Some isolates were not amplified using 16S rRNA gene sequencing; therefore, *Rhodotorula* (one strain) and *Pichia* (three strains) were identified from the gills of *Chionoecetes* sp. using ITS gene sequencing.

### Enumeration of total cultivable bacteria

**Figure 2:**
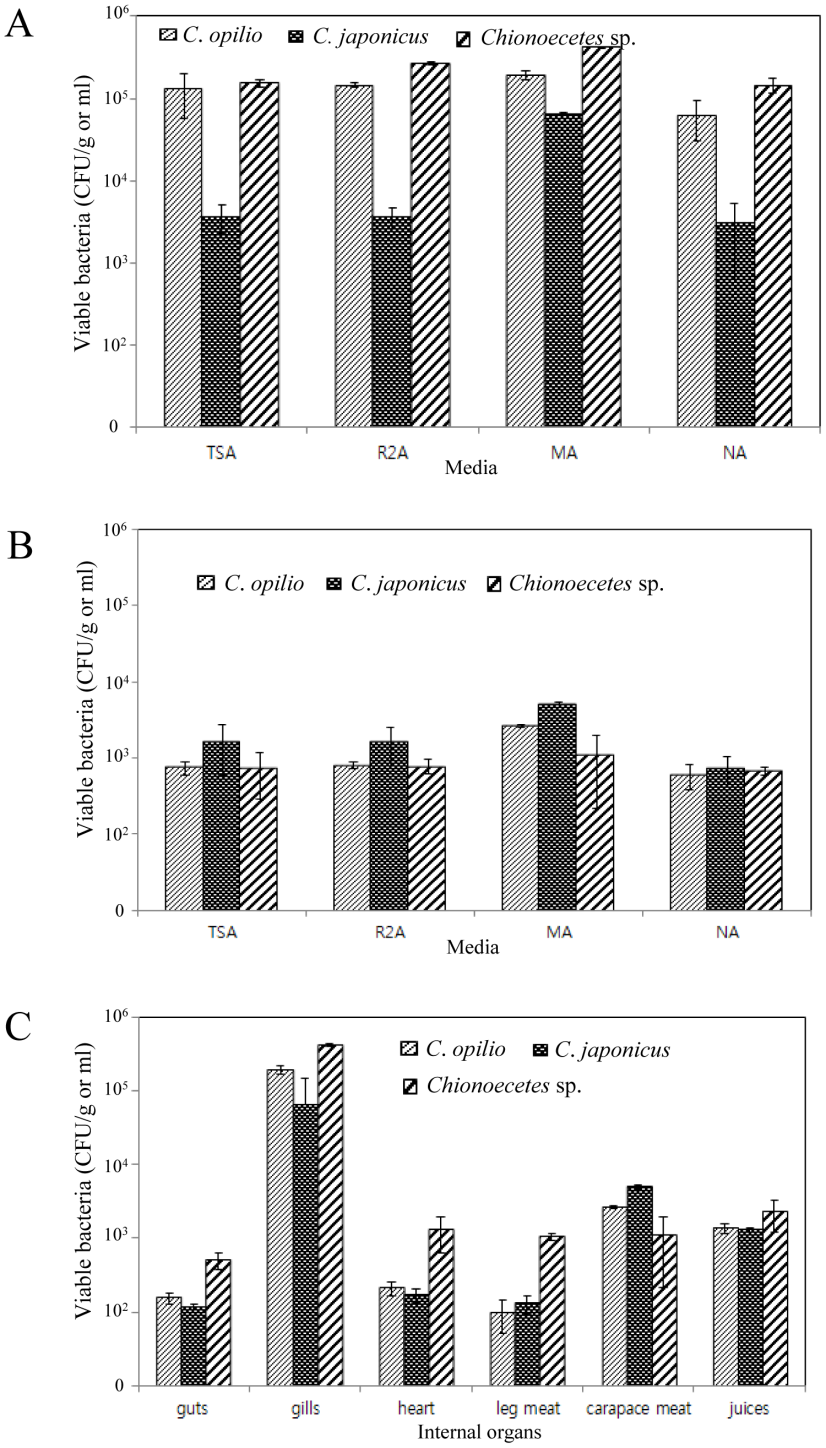
Enumeration of total cultivable snow crab-dwelling bacteria at each site, in each crab species. Snow crab gill-dwelling bacteria on four different solid media (A), carapace meat-dwelling bacteria (B), total colony counts of bacteria at each site in each snow crab on Marine Agar media (C). D: guts; J: carapace juices; G: gills; H: heart; LS: leg meat; S: carapace meat.

As shown in Figure 2, aerobic bacteria counts numbered from 10^3^ to 10^5^ cells/g in gills and from 10^2^ to 10^3^ cells/g in carapace meat in the three snow crab species on TSA, R2A, MA, and NA media, whereas no aerobic bacteria or other prokaryotes appeared on YPD or PDA media (Figures 2A and 2B). In comparison with the other organs, gills of the three crab species contained relatively high aerobic bacterial populations (up to 10^5^) on TSA, R2A, MA and NA (Figures 2A and 2C).

### Enumeration of antibiotic-resistant bacteria

**Figure 3:**
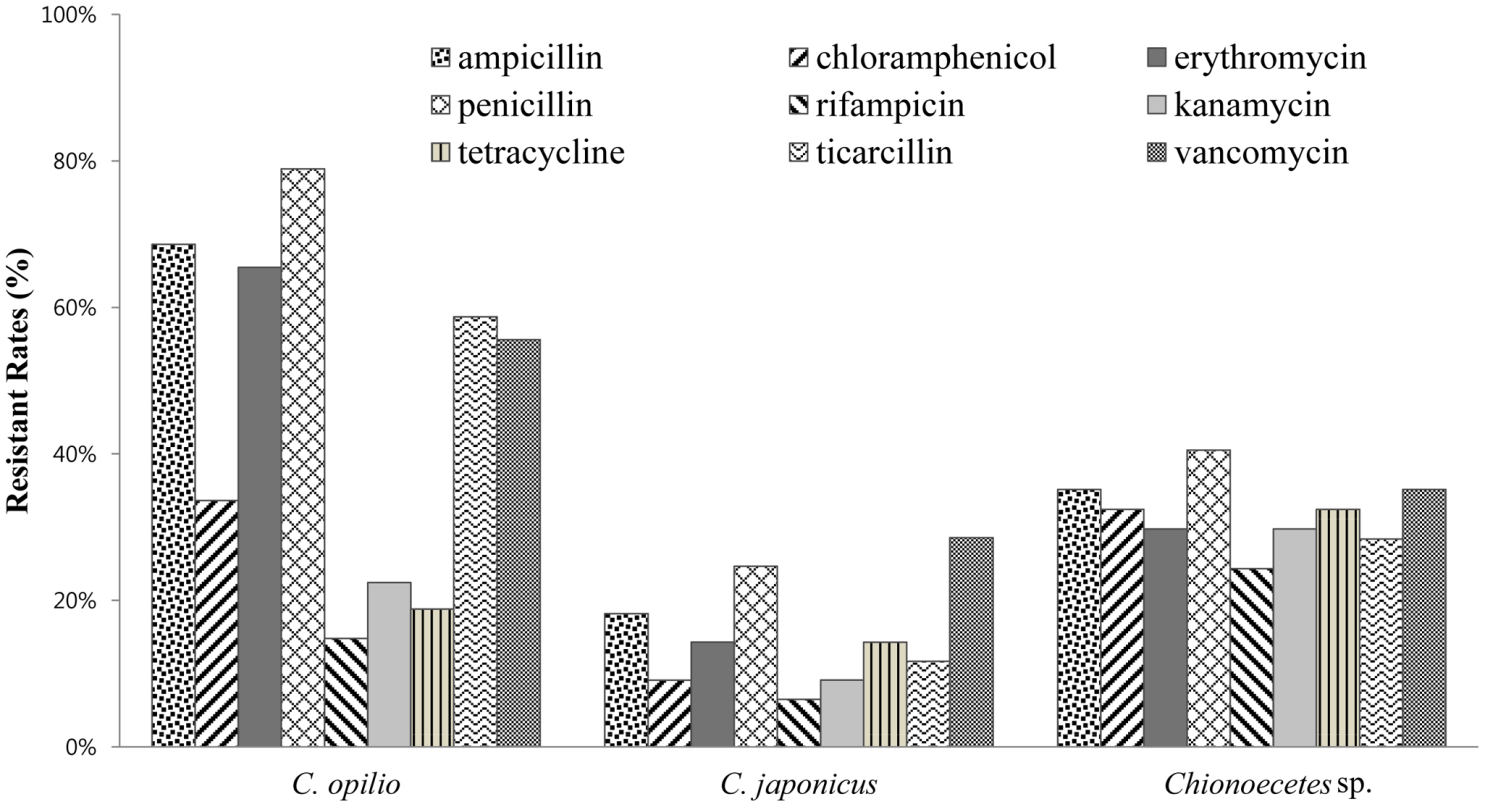
Antibiotic resistance rates to nine different antibiotics in three species of snow crab. Am: ampicillin; C: chloramphenicol; E: erythromycin; P: penicillin; RA: rifampicin; K: kanamycin; Te: tetracycline; Tic: ticarcillin; Va: vancomycin.

More than 50% of the 221 *C. opilio* isolates were resistant to ampicillin, erythromycin, penicillin, ticarcillin, and vancomycin. Isolates from *C. japonicus* and *Chionoecetes* sp. were resistant to the nine antibiotics tested, representing a 15% and 30% resistance ratio respectively (Figure 3 and Table 2). In *C. opilio*, *Pseudomonas*, *Acinetobacter*, *Enterobacter*, *Psychrobacter*, *Stenotrophomonas*, and *Lactobacillus* spp. were resistant to the nine antibiotics tested (19 isolates); *Pseudomonas*, *Acinetobacter*, and *Stenotrophomonas* spp. were resistant to seven or eight of the nine antibiotics (12 isolates; Figures S1a, S1b, S1c and Table 2). The MDR bacteria identified in our study were affiliated with *Agreia* and *Psychrobacter*; two isolates from *C. japonicus* (red-tanner crab; Figure S1f and Table 2) and nine genera (14 isolates) from *Chionoecetes* sp. were affiliated with *Shewanella*, *Rhodococcus*, *Agrococcus*, *Leifsonia*, *Deinococcus*, *Staphylococcus*, and *Agreia* bacteria, and with *Rhodotorula* and *Pichia* yeasts (Figure S1g and Table 2).

**Table 2:** The media used to isolate bacterial strains displayed the relationship of multidrug resistance (MDR) in bacteria from snow crab.

			Medium					
Snow Crab	Taxa	Phyla	NA1)	YPD2)	PDA3)	R2A4)	TSA5)	MA6)
	*Pseudomonas*			1 (1)#			1 (0)	5 (2)
	*Acinetobacter*			6 (6)				4 (3)
	*Stenotrophomonas*			1 (1)	1 (0)			7 (1)
*C. opilio*	*Psychrobacter*	γ7) (GN)11)						1 (1)
(19/31)*	*Enterobacter*							1 (1)
	*Leclercia*							1 (1)
	*Lactobacillus*	B8)(GP)12)			1 (1)		1 (1)	
*C. japonicus*	*Salinibacter*	A9) (GP)	1 (1)					
(2/2)	*Psychrobacter*	γ (GN)	1 (1)					
	*Agrococcus*		2 (2)					
	*Salinibacter*	A (GP)	1 (1)			1 (1)	1 (1)	
*Chionoecetes* sp.	*Rhodococcus*		2 (2)			1 (1)		
(14/14)	*Deinococcus*	D10) (GP)	1 (1)					
	*Staphylococcus*	B (GP)				1 (1)		
	*Shewanella*	γ (GN)					1 (1)	
	*Pichia*	Yeast	1 (1)	1 (1)				
	*Rhodotorula*	Yeast					1 (1)	

* (Numbers of nine antibiotic-resistant bacteria among more than seven antibiotic-resistant bacteria).

# Parentheses indicated all resistant bacteria to tested nine antibiotics.

1) NA : Nutrient Agar,

2) YPD : Yeast Extract Peptone Dextrose,

3) PDA : Potato Dextrose Agar.

4) R2A Agar : Reasoner's 2A agar,

5) TSA ; Tryptic Soy Agar,

6) MA : Marine Agar.

7) γ : gamma Proteobacteria,

8) B : Bacilli,

9) A : Actinobacteria,

10) D : Deinococcus,

11) GN : gram-negative bacteria,

12) GP : gram-positive bacteria.

### Phylogenetic distribution

Phylogenetic analysis revealed that isolates within *Pseudomonas* and *Stenotrophomonas* were grouped separately, representing 66 and 62 isolates from *C. opilio* (snow crab, C), while two and three isolates from *C. japonicus* (red-tanner crab, CJ) and *Chionoecetes* sp. (Neodo-Daege, B) were represented (Figures S1a and S1c). For *Acinetobacter* spp., 49, 14, and one isolate were found in *C. opilio*, *C. japonicus*, and *Chionoecetes* sp., respectively (Figure S1b). In *C. japonicus* three clusters formed with high similarly to *A. johnsonii* DSM 6963, *A. haemolyticus* DSM 6962, and *A. guillouiae* ATCC 11171 (Figure S1b). Up to 26 *Bacillus* spp. were isolated from *Chionoecetes* sp. and nine *Bacillus* spp. isolates were found in each of *C. opilio* and *C. japonicus* (Figure S1d). These isolates showed similarity with several comparative groups, suggesting that the relationship between the species of *Chionoecetes* and the isolated *Bacillus* spp. was not strong (Figure S1d). Other actinobacteria were found in *Chionoecetes* sp. and *C. japonicus* (34 and 21 isolates, respectively). Interestingly, 10 isolates of *Salinibacterium* from *C. japonicus* were clustered together (Figures S1f and S1g). Isolates from *Chionoecetes* sp. were affiliated with *Microbacterium* (eight isolates), *Rhodococcus* (nine isolates), and *Deinococcus* (two isolates; Figure S1g). Proteobacterial taxa were affiliated with 16 isolates of γ-proteobacteria from a total of 17 isolates from *C. japonicus* (Figure S1f). Isolates from *C. opilio* were affiliated with α- (seven isolates), β- (five isolates), and γ-proteobacteria (nine isolates; Figure S1e). Bacteroidetes taxa from *C. japonicus* were affiliated with *Flavobacterium* (two isolates) and *Chryseobacterium* (one isolate) in *Chionoecetes* sp. Bacilli, excluding the *Bacillus*, were isolated from *C. opilio* (five isolates), *C. japonicas* (five isolates), and *Chionoecetes* sp. (six isolates). Specifically, *Exiguobacterium* was isolated from *C. japonicus* (Figure S1f).

### PCR detection of antibiotic resistance genes

We tested the antibiotic multiresistant bacteria, i.e., *Acinetobacter* spp., *Leclercia* sp., *Pseudomonas* spp., *Stenotrophomonas* spp., *Lactobacillus* spp., and *Bacillus* sp. for the detection of antibiotic resistance genes, i.e., *bla_SHV_*, *bla_OXA_*, and *bla_TEM_* as ampicillin resistance genes; *catA1*, *catA2*, *catA3*, and *catB3* as chloramphenicol resistance genes; *ermA*, *ermB*, and *mefA* genes as erythromycin resistance genes; *pbp2a* as penicillin resistance gene; *aphA-3* as kanamycin resistance genes; *tetA*, *tetB*, *tetC*, and *tetG* as tetracycline resistance genes; and *vanA* and *vanB* as vancomycin resistance genes. The *catA1* gene was detected in all the tested bacteria. However, resistance genes against ampicillin, erythromycin, penicillin, and kanamycin were not found in the tested bacterial isolates. Interestingly, *Leclercia* sp. possessed *catB* and *Pseudomonas* sp. possessed *tetB*. Vancomycin resistance gene, *vanB* was detected in *Pseudomonas* spp. and *Stenotrophomonas* spp (Table 3). Sequencing analysis of the PCR products showed that the sequences of *catA1* gene were identical, with 100% nucleotide homology in the tested isolates except for the gene of *Leclercia* spp. The *catA1* sequence of *Acinetobacter* sp. C-G-MA6 (AB826491) and *Pseudomonas* sp. C-D-MA7 (AB826493) showed that the gene represented 99% and 100% nucleotide identity to an antibiotic resistance gene of *Klebsiella pneumonia* subsp. *pneumonia* KPX plasmid pKPX-1 DNA. Moreover, amino acid sequences translated from the nucleotide sequences of the PCR products showed 100% identity with the amino acid sequence of chloramphenicol acetyltransferase. This result indicates that *catA1* gene is derived from the chloramphenicol resistance gene detected in several pathogenic bacteria.

**Table 3:** The specific resistance of the multidrug resistance (MDR) bacterial strains.

	Isolates	Genera	Group	Resistance phenotype	PCR detection
	C-D-PYD4	*Acinetobacter*	GN1)	Am, Chl, Em, Pen, Rif, Km, Tet, Tc, Van	
	C-G-MA6	*Acinetobacter*	GN	Am, Chl, Em, Pen, Rif, Km, Tet, Tc, Van	*catA1*
	C-G-MA4	*Acinetobacter*	GN	Am, Chl, Em, Pen, Rif, Km, Tet, Tc, Van	
	C-G-PYD9	*Acinetobacter*	GN	Am, Chl, Em, Pen, Rif, Km, Tet, Tc, Van	
	C-J-PYD3	*Acinetobacter*	GN	Am, Chl, Em, Pen, Rif, Km, Tet, Tc, Van	
	C-LS-MA1	*Acinetobacter*	GN	Am, Chl, Em, Pen, Rif, Km, Tet, Tc, Van	
	C-LS-PYD3	*Acinetobacter*	GN	Am, Chl, Em, Pen, Rif, Km, Tet, Tc, Van	
	C-S-PYD1	*Acinetobacter*	GN	Am, Chl, Em, Pen, Rif, Km, Tet, Tc, Van	*catA1*
	C-S-PYD3	*Acinetobacter*	GN	Am, Chl, Em, Pen, Rif, Km, Tet, Tc, Van	
	C-S-MA2	*Acinetobacter*	GN	Am, Chl, Em, Pen, Tet, Tc, Van	
	C-G-MA1	*Leclercia*	GN	Am, Chl, Em, Pen, Rif, Km, Tet, Tc, Van	*catA1, catB*
	C-D-MA4	*Pseudomonas*	GN	Am, Chl, Em, Pen, Rif, Km, Tet, Tc, Van	*catA1, tetB*
	C-LS-PYD4	*Pseudomonas*	GN	Am, Chl, Em, Pen, Rif, Km, Tet, Tc, Van	*catA1*
	C-LS-MA4	*Pseudomonas*	GN	Am, Chl, Em, Pen, Rif, Km, Tet, Tc, Van	
***C. opilio***	C-S-MA1	*Pseudomonas*	GN	Am, Chl, Em, Pen, Rif, Tet, Tc, Van	
	C-S-MA7	*Pseudomonas*	GN	Am, Chl, Em, Pen, Rif, Tet, Tc, Van	
	C-D-MA7	*Pseudomonas*	GN	Am, Chl, Em, Pen, Rif, Tet, Tc, Van	*catA1, vanB*
	C-D-TSA1	*Pseudomonas*	GN	Am, Chl, Em, Pen, Rif, Te, Van	
	C-G-MA5	*Stenotrophomonas*	GN	Am, Chl, Em, Pen, Rif, Km, Tet, Tc, Van	*catA1, vanB*
	C-S-PYD2	*Stenotrophomonas*	GN	Am, Chl, Em, Pen, Rif, Km, Tet, Tc, Van	*catA1*
	C-LS-MA2	*Stenotrophomonas*	GN	Am, Em, Pen, Km, Tet, Tc, Van	
	C-LS-MA5	*Stenotrophomonas*	GN	Am, Em, Pen, Km, Tet, Tc, Van	
	C-LS-MA7	*Stenotrophomonas*	GN	Am, Em, Pen, Km, Tet, Tc, Van	
	C-J-MA2	*Stenotrophomonas*	GN	Am, Em, Pen, Km, Tet, Tc, Van	
	C-J-MA5	*Stenotrophomonas*	GN	Am, Em, Pen, Km, Tet, Tc, Van	
	C-G-MA2	*Stenotrophomonas*	GN	Am, Em, Pen, Km, Tet, Tc, Van	*catA1*
	C-S-PDA4	*Stenotrophomonas*	GN	Am, Em, Pen, Km, Tet, Tc, Van	
	C-J-MA7	*Enterobacter*	GN	Am, Chl, Em, Pen, Rif, Km, Tet, Tc, Van	
	C-G-MA3	*Psychrobacter*	GN	Am, Chl, Em, Pen, Rif, Km, Tet, Tc, Van	
	C-G-TSA3	*Lactobacillus*	GP2)	Am, Chl, Em, Pen, Rif, Km, Tet, Tc, Van	
	C-LS-PDA4	*Lactobacillus*	GP	Am, Chl, Em, Pen, Rif, Km, Tet, Tc, Van	*catA1*
***C. japonicus***	CJ-G-NA9	*Salinibacterium*	GP	Am, Chl, Em, Pen, Rif, Km, Tet, Tc, Van	
	CJ-S-NA3	*Psychrobacter*	GN	Am, Chl, Em, Pen, Rif, Km, Tet, Tc, Van	
	B-G-NA3	*Rhodococcus*	GP	Am, Chl, Em, Pen, Rif, Km, Tet, Tc, Van	
	B-G-NA8	*Rhodococcus*	GP	Am, Chl, Em, Pen, Rif, Km, Tet, Tc, Van	
	B-G-R2A1	*Rhodococcus*	GP	Am, Chl, Em, Pen, Rif, Km, Tet, Tc, Van	
	B-G-R2A7	*Rhodococcus*	GP	Am, Chl, Em, Pen, Rif, Km, Tet, Tc, Van	
	B-G-NA4	*Agrococcus*	GP	Am, Chl, Em, Pen, Rif, Km, Tet, Tc, Van	
	B-G-NA10	*Agrococcus*	GP	Am, Chl, Em, Pen, Rif, Km, Tet, Tc, Van	
***Chionoecetes*** ** sp.**	B-G-NA5	*Leifsonia*	GP	Am, Chl, Em, Pen, Rif, Km, Tet, Tc, Van	
	B-G-R2A5	*Leifsonia*	GP	Am, Chl, Em, Pen, Rif, Km, Tet, Tc, Van	
	B-G-NA11	*Deinococcus*	GP	Am, Chl, Em, Pen, Rif, Km, Tet, Tc, Van	
	B-G-R2A2	*Staphylococcus*	GP	Am, Chl, Em, Pen, Rif, Km, Tet, Tc, Van	
	B-G-TSA8	*Shewanella*	GN	Am, Chl, Em, Pen, Rif, Km, Tet, Tc, Van	
	B-G-NA7	*Pichia*	Yeast	Am, Chl, Em, Pen, Rif, Km, Tet, Tc, Van	
	B-G-PYD12	*Pichia*	Yeast	Am, Chl, Em, Pen, Rif, Km, Tet, Tc, Van	
	B-G-TSA1	*Rhodotorula*	Yeast	Am, Chl, Em, Pen, Rif, Km, Tet, Tc, Van	

1) GN : Gram negative bacteria,

2) GP : Gram positive bacteria,

Abbreviations used: Am, ampicilline; Chl, chloramphenicol; Em, erythromycin; Pen, penicillin; Rif, rifampicin; Km, kanamycin;

Tet, tetracycline; Tc, ticarcillin; Van, vancomycin.

The underline indicates tested isolates for detection of MDR genes by PCR.

GN : gram-negative bacteria, GP : gram-positive bacteria.

### Nucleotide sequence accession numbers

All sequences were deposited in GenBank under the following accession numbers: HM755454–HM755674 (*C. opilio*), HM584223–HM584298 (*C. japonicus*), HM629343–HM629422, and HM588762–HM588765 (*Chionoecetes* sp.).

## Discussion

Until recently, the study of *Chionoecetes* was conducted mainly by artificial cultivation for the examination of disease [23], [24]. In contrast, the present study was performed to characterize the types of microbiota within *Chionoecetes*, as little is known about microbial dynamics within *Chionoecetes* [23], [24]. In this study, we provided ratios of antibiotic resistance and microbial community distribution in three *Chionoecetes* species. Our results indicate that future microbial studies of *Chionoecetes* in their natural ecosystems are necessary to assess and monitor potential human risk.

This study revealed four genera prevalent in *Chionoecetes*: *Pseudomonas*, *Acinetobacter*, *Stenotrophomonas*, and *Bacillus* (Figure S1). Microbial diversity was high in the gills of *C. opilio* (Figure 2C). Schuwerack et al. [25] reported that bacterial colonies enmeshed in polysaccharide-like films produced indentations in the gill cuticular surfaces and dissociation of microvillus membranes at the basal zone of epithelial cells of gill lamellae of the fresh crab *Potamonautes warren*.

The yeasts *Rhodotorula* and *Pichia, which* were identified from *Chionoecetes* sp. *Rhodotorula*, *Cryptococcus*, *Torulopsis*, *Candida*, *Trichosporon*, and *Aureobasidium*, have previously been isolated from the meat of Dungeness (*Cancer magister*) and King crabs (*Paralithodes camtschatica*) [26]. The discovery of yeast in *C. opilio* in the present study, as well as in Dungeness and King crabs [26], suggests that future ecological studies of yeast populations will be necessary as well.

While several studies show conclusively that antibiotic resistance is a natural phenomenon that predates the modern selective pressures of clinical antibiotics and agricultural use of antibiotics [27]–[33], human activity has probably increased the prevalence of MDR bacteria in air, soil, and marine and freshwater ecosystems. Most antibiotic resistance genes are acquired through horizontal gene transfer [8]. In this study, MDR bacteria from *Chionoecetes* demonstrated antibiotic resistance in nonclinical environments, suggesting an ecological role for antibiotics that warrants additional investigation.

Some *Bacillus* spp. (e.g., *Bacillus cereus*) are ubiquitous in nature and constitute a major portion of the microbial populations in contaminated food, causing food spoilage and poisoning to the detriment of the consumers [34]. Two *Bacillus* species are considered medically significant: *B. anthracis*, which causes anthrax, and *B. cereus*, which causes a food-borne illness [34]. Because we found *Bacillus* spp. in all *Chionoecetes*, the incidence and survival of *Bacillus* spp. is thought to be controlled by cooking *Chionoecetes* at high temperatures prior to consumption.

In terms of antibiotic-resistant *Acinetobacter* spp., the bacteria were found in *C. opilio* and *C. japonicus*; infections generally occur in hospitalized patients with weakened immune systems. Therefore, understanding antibiotic resistance in *C. opilio* isolates is clinically important for cases involving multidrug resistance (MDR) [35]. In this study, 54 isolates of *Acinetobacter* spp. were isolated from *C. opilio*, more than 60% of which were resistant to at least one of the antibiotics tested. Antibiotic resistance was high not only for the dominant *Pseudomonas*, *Acinetobacter*, and *Stenotrophomonas* (γ-proteobacteria), but was also high regardless of whether bacterial strains were gram negative or gram positive. γ-Proteobacteria demonstrated high antibiotic resistance to isolates from *C. opilio*, while the actinobacteria of *Chionoecetes* sp. were resistant to all of the nine antibiotics tested (Table 2). Here, we revealed that actinobacteria were commonly isolated from *Chionoecetes* sp., including many multidrug-resistant strains (Table 2). Moreover, clinical reports of secondary urinary or respiratory infections by *Pseudomonas* and *Enterobacter* spp. have been presented [36], [37]. While isolates of snow crabs rarely infect respiratory organs or the skin, proper heating of food prepared from *Chionoecetes* must be ensured in order to protect against infection.

Similar results for MDR bacteria have been observed in shrimp [38]–[40], chicken [41]–[43], fruit [42], vegetables [42], pork [42], [43], salad [44], drinking water [45], fish [46]–[48], and fish farms [49], [50]. These commonalities also suggest that MDR bacteria should be investigated from many samples following standard methods described by the National Committee for Clinical Laboratory Standardization [51]. It is also necessary to replicate studies of microbiota and the inhibition zone diameter.

The results of the present genetic study showed that the *catA1* gene is widespread in many bacteria (Table 3). These data indicated that this gene moved between species via horizontal gene transfer. However, whether the multiresistance of the bacteria could be derived from intrinsic characteristics of bacteria or from unknown mechanisms (e.g., uncharacterized specific genes and dissemination through unknown transposable elements) is an open question. Therefore, we suggest that further studies are necessary to elucidate whether the resistance gene of snow crabs is intrinsic or arises from horizontal gene transfer between the environmental and pathogenic resistomes. Additional research is required to determine how resistance genes become incorporated into a range of bacteria species. In the future, it is essential that the implications of MDR for human consumption of snow crabs be entirely understood and that the penetration of antibiotic resistance into natural environments be prevented.

In summary, we revealed for the first time a high level of microbial infiltration or inclusion in the internal organs of three *Chionoecetes* species. In addition, we isolated 381 microbial strains from three species of *Chionoecetes* spp.; unexpectedly, microbes with antibiotic resistance are widely distributed throughout the internal organs of wild, commercial snow crabs. In the future, additional research on antibiotic resistance and its mechanism and on microbial dynamics in the fishery industry will enhance further understanding of the clinical and ecological implications of these results.

## Supporting Information

Figure S1 **Phylogenetic tree of dominant bacteria in three species of snow crab.** (a): *Pseudomonas* spp., (b): *Acinetobacter* spp., (c): *Stenotrophomonas* spp., (d): *Bacillus* spp., (e): the other of *C. opilio*, (f): the other of *C. japonicus* crab, (g): *Chionoecetes* sp. Bootstrap values represent the percentage of 1,000 replicates. Box indicates resistance to more than seven antibiotics. Dark star symbol (★) in boxes indicates resistance against tested nine antibiotics, light star symbol (☆) against eight antibiotics, and box without symbol against seven antibiotics. C: *Chionoecetes opilio*, CJ: *C. japonicus*, B: *Chionoecetes* sp.
(TIF)
